# Physiological Characteristics of Cultivated Tepary Bean (*Phaseolus acutifolius* A. Gray) and Its Wild Relatives Grown at High Temperature and Acid Soil Stress Conditions in the Amazon Region of Colombia

**DOI:** 10.3390/plants11010116

**Published:** 2021-12-31

**Authors:** Juan Carlos Suárez, Amara Tatiana Contreras, José Alexander Anzola, José Iván Vanegas, Idupulapati M. Rao

**Affiliations:** 1 Programa de Ingeniería Agroecológica, Facultad de Ingeniería, Universidad de la Amazonia, Florencia 180001, Colombia; amaratatis18@gmail.com (A.T.C.); 1993anzola@gmail.com (J.A.A.); vanegas.agroeco@gmail.com (J.I.V.); 2 Programa de Maestría en Sistemas Sostenibles de Producción, Facultad de Ingeniería, Universidad de la Amazonia, Florencia 180001, Colombia; 3 Grupo de Investigaciones Agroecosistemas y Conservación en Bosques Amazónicos-GAIA, Centro de Investigaciones Amazónicas CIMAZ Macagual César Augusto Estrada González, Florencia 180001, Colombia; 4 International Center for Tropical Agriculture (CIAT), Km 17 Recta Cali-Palmira, Cali 763537, Colombia; i.rao@cgiar.org

**Keywords:** heat tolerance, energy use, chlorophyll fluorescence, agronomic characteristics, yield

## Abstract

Common bean (*Phaseolus vulgaris* L.) is sensitive to different types of abiotic stresses (drought, high temperature, low soil fertility, and acid soil), and this may limit its adaptation and consequently to its yield under stress. Because of this, a sister species, tepary bean (*Phaseolus acutifolius* A. Gray), has recently gained attention in breeding for improved abiotic stress tolerance in common bean. In this study, we evaluated the adaptation of 302 accessions of tepary bean (*Phaseolus acutifolius* A. Gray) and its wild relatives (grouped in four types of tepary bean genetic resource: cultivated, acutifolius regressive, acutifolius wild, tenuifolius wild) when grown under high temperature and acid soil conditions with aluminum toxicity in the Amazon region of Colombia. Our objective was to determine differences among four types of tepary bean genetic resource in their morpho-phenological, agronomic, and physiological responses to combined high temperature and acid soil stress conditions. We found that cultivated *P. acutifolius* var acutifolius presented a greater number of pods per plant, as well as larger seeds and a greater number of seeds per pod. Some traits, such as root biomass, days to flowering and physiological maturity, specific leaf area, and stomatal density, showed significant differences between types of tepary bean genetic resource, probably contributing to difference in adaptation to combined stress conditions of high temperature and acid soil conditions. The photochemical quenching (qP) was higher in cultivated *P. acutifolius* var. acutifolius, while energy dissipation by non-photochemical quenching (NPQ) in the form of heat and the coefficient of non-photochemical dissipation (qN) were higher in acutifolius regressive and tenuifolius wild accessions. We have identified 6 accessions of cultivated and 19 accessions of tenuifolius wild that exhibited grain yields above 1800 kg ha^−1^. These accessions could be suitable to use as parents to improve dry seed production of tepary bean under combined stress conditions of high temperature and acid soil.

## 1. Introduction

The effects of climate change are rapidly impacting ecosystems around the world [1] by generating alterations related to increased global average temperatures, changes in precipitation patterns [2], and increased CO_2_ levels [3]. Such alterations have placed increased pressure on agriculture due to reduced water availability, which limits the use of agricultural land for food production [3]. It has been estimated that agriculture by 2050 will need to produce 60% to 100% more food than is currently produced [1], becoming a major challenge to address food security in the coming decades [4]. Likewise, these dramatic near-term climate change scenarios give rise to predictions of yield reduction in crops, especially those with greater abiotic stress sensitivity, such as common bean [5].

This has led to the identification and characterization of new sources of abiotic stress tolerance as one of the most urgent requirements to cope with the effects of climate change on crop production [6]. Among the food crops, the common bean (*Phaseolus vulgaris* L.) stands out due to its global importance as the most consumed food legume, and it makes a major nutritional contribution to the human diet [7]. However, its high sensitivity to different types of abiotic stresses (low soil fertility, high temperatures, drought and high rainfall) may represent a limitation for its adaptation and, consequently, in its yield [8,9,10,11]. Because of this, a sister species, such as tepary bean (*Phaseolus acutifolius* A Gray.), has recently gained attention in breeding efforts for improving abiotic stress tolerance in common bean [12,13,14].

Tepary bean has been domesticated as a crop, and it has been produced from the western Mexican states of Jalisco and Colima to the southwestern United States [15]. It exhibits many unique and favorable genetic attributes, such as disease resistance [16,17], and possesses higher levels of heat and drought tolerance compared to common bean [12,13,14,18], as well as tolerance to low soil fertility [19]. Tepary bean exhibits different mechanisms of stress tolerance, including deep rooting to avoid dehydration, small leaves, and stomatal control to reduce water use [20,21]. In addition to the above, tepary bean seed contains about 24% as protein; fatty acid composition of 33% as saturated fatty acids; 67% as unsaturated fatty acids (24% as monounsaturated fatty acids; 42% as polyunsaturated fatty acids); and also with high mineral content (Ca, Mg, K, Fe, Cu, Zn, Mn, S) [22,23,24]. These nutritional attributes make it as a valuable crop for dry environments where crop yields are generally low [25].

Tepary bean is part of the tertiary gene pool of the common bean [12], and, thus far, two botanical varieties are recognized: (i) *P. acutifolius* var. acutifolius with oval-lanceolate leaflets; and (ii) *P. acutifolius* var. tenuifolius with linear-lanceolate leaflets [26], of which four types of genetic resources are reported in tepary varieties, such as cultivated, wild, and regressive acutifolius and wild tenuifolius [27].

Although detailed studies exist on comparing wild and cultivated ancestors [28,29] of common bean, tepary bean has little comparable information among its varieties that are grouped into four types of genetic resource [25,26]. In addition, due to the differences between *P. acutifolius* and the other *Phaseolus* species (*P. vulgaris*, *P. lunatus*, *P. coccineus*, and *P. dumosus*) [30], there is no information on its adaptive responses to more humid and warmer tropical regions of Central America, the Andes, and the Amazon basin [26]. The distribution of tepary bean is limited mainly to its domestication center in the dry subtropical Pacific slopes [31].

Most of the regions where beans are currently grown are located in the tropics, and it is estimated that almost 50% of potentially arable land is acidic [32]. For example, the Latin American region alone accounts for 40% of acidic soils worldwide, which represents a limitation for common bean cultivation [33]. In the Amazon region, the adaptation of advanced lines of common bean under acid soils with low fertility and high temperature conditions has been studied [10,19,34] to identify materials with adaptability, thus being able to improve the diet of small farmers in rural areas. However, the available germplasm collection of *Phaseolus acutifolius* accessions was not evaluated to identify materials that can adapt to the combined stress of high temperature and low fertility acid soils with high aluminum (Al) toxicity.

Genotypic differences among tepary bean accessions in terms of morphological, agronomic and physiological attributes were not evaluated. At the physiological level, adaptation to high temperature and acid soil stress conditions could be evaluated using chlorophyll fluorescence (Chl_a_), which helps to quantify genotypic differences in using energy in response to the level of stress, particularly the energy absorbed by PSII distributed to the photochemical, heat dissipation, or unregulated pathways [34]. Thus, the objective of this study was to evaluate the differences in morpho-physiological and agronomic adaptation of cultivated tepary bean (*Phaseolus acutifolius* A. Gray) and its three relatives (acutifolius regressive, acutifolius wild and tenuifolius wild) under the combined stress condition of high temperature and acid soil stress in the Amazon region of Colombia. We tested the hypothesis that tepary bean accessions that combine a higher carbon assimilation capacity with a better mobilization of photoassimilates to developing pods and seeds under conditions of high temperature and acid soil stress are better adapted to the Colombian Amazon. The results from this study are important for the improvement of the common bean, since crosses with better adapted accessions of tepary bean can increase yield of common bean under the combined acid soil and high temperature stress conditions.

## 2. Results

### 2.1. Development of Different Vegetative Organs under High Temperature and Acid Soil Stress Conditions

**Figure 1:**
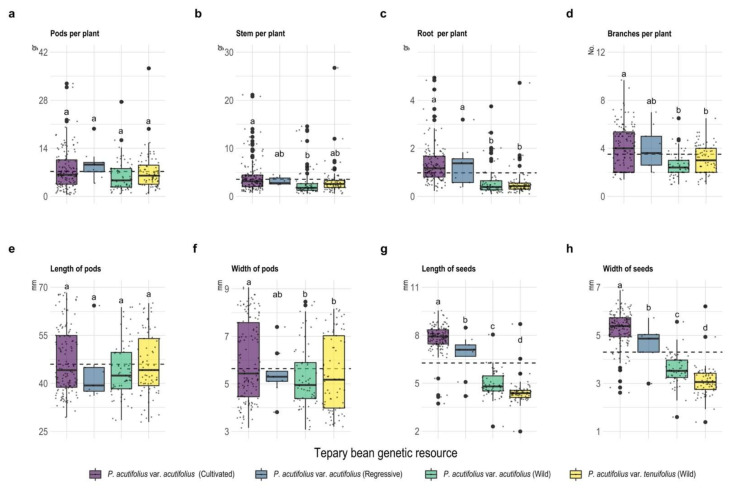
Box plots of the scatter plot for agronomic characteristics at the plant (**a**–**d**), pod (**a**,**e**,**f**), and seed (**g**,**h**) levels among the tepary bean genetic resource. Dotted line means the overall mean for each variable. Different letters between tepary bean genetic resource indicate different means (*p* < 0.05).

Results on evaluation of different accessions of *Phaseolus acutifolius* under high temperature and acid soil conditions showed significant differences among the four types of accessions at the pod (width) and seed (length and width), as well as root, level (Figure 1, *p* < 0.001). Pod production per plant averaged 7.26 ± 0.3 g with values above and below the mean for the cultivated and wild type of *P. acutifolius* var. acutifolius with 9.36 ± 1.5 and 5.91 ± 0.59 g. In the case of root biomass per plant, both the cultivated and regressive type of *P. acutifolius* var. acutifolius accumulated 1.34 ± 0.07 and 1.30 ± 0.29 g, respectively, and these values were higher than the mean (0.98 ± 0.05 g). Regarding seed size in relation to length and width, from largest to smallest, respectively, we found the following order: cultivated *P. acutifolius* var. acutifolius (7.83 ± 0.07 and 5.27 ± 0.06 mm in length and width, respectively), followed by *P. acutifolius* var. acutifolius regressive (6.75 ± 0.44 and 4.61 ± 0.26 mm), *P. acutifolius* var. acutifolius wild (4.95 ± 0.1 and 3.58 ± 0.08 mm), and, lastly, *P. acutifolius* var. tenuifolius wild (4.42 ± 0.09 and 3.09 ± 0.07 mm).

**Figure 2:**
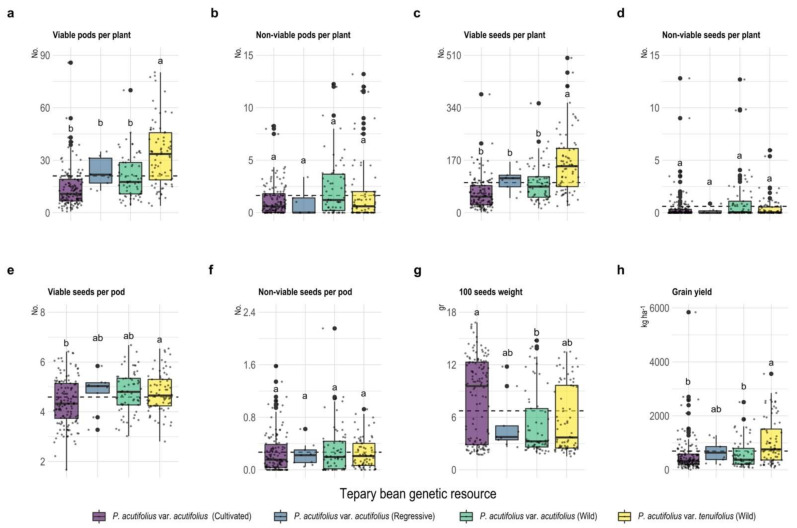
Box plots of the scatter plot for agronomic grain characteristics in terms of viable and non-viable seeds per plant (**a**–**d**), viable and non-viable seeds per pod (**e**,**f**), 100 seeds weight (**g**) and grain yield (**h**) of tepary bean genetic resource. Dotted line means the overall mean for each variable. Different letters between tepary bean genetic resource indicate different means (*p* < 0.05).

Regarding the non-viability of pods and seeds per plant and seeds per pod, we found no differences among the four types of accessions (*p* > 0.05, Figure 2). However, some type of accessions presented a higher production of viable pods per plant, viable seeds per plant, and viable seeds per pod (*p* < 0.001) above the mean (21.0 ± 0.96 pods per plant, 97.72 ± 4.82 seeds per plant, 4.55 ± 0.06 seeds per pod), with a production of 33.8 ± 2.18 pods per plant, 161.62 ± 11. 7 seeds per plant, and 4.76 ± 0.09 seeds per pod for wild *P. acutifolius* var. tenuifolius, followed by regressive *P. acutifolius* var. acutifolius (22.89 ± 2.75, 106.57 ± 12, 4.73 ± 0.27), *P. acutifolius* var. acutifolius wild (20.71 ± 1.62, 96.5 ± 7.86, 4.72 ± 0.11), and *P. acutifolius* var. acutifolius cultivated (14.1 ± 0.98, 62.88 ± 4.51, 4.35 ± 0.09). As for 100-seed weight, it ranged from 1.54 to 16.83 g with a mean of 6.74 ± 0.27 g, with *P. acutifolius* var. acutifolius cultivated being the type with the highest mass (8.09 ± 0.41 g). Grain yield (GY) in *P. acutifolius* accessions ranged from 6.86 to 5841.23 kg ha^−1^, where accessions within the wild *P. acutifolius* var. tenuifolius group had the highest yields, exceeding 1000 kg ha^−1^ (Figure 2).

### 2.2. Morphological and Phenological Traits

**Figure 3:**
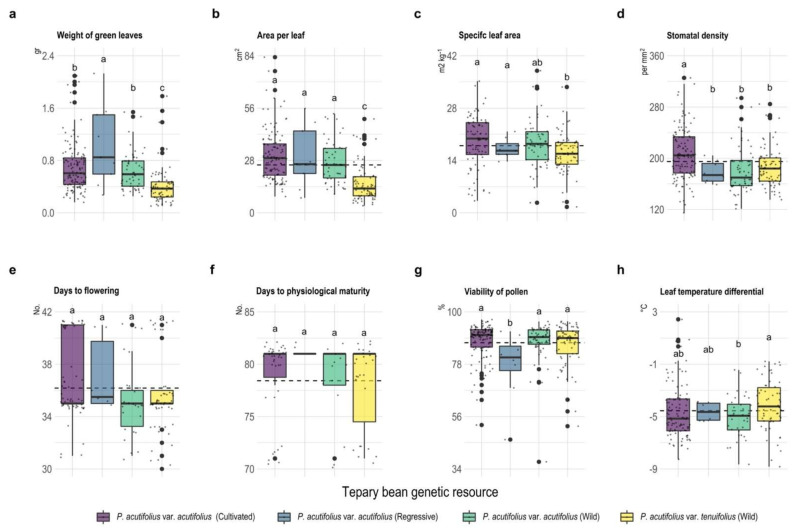
Box plots of the scatter plot for morphological (**a**–**d**), phenological (days to flowering (**e**) and days to physiological maturity (**f**)) and physiological (**g**,**h**) characteristics of tepary bean genetic resource. Dotted line means the overall mean for each variable. Different letters between tepary bean genetic resource indicate different means (*p* < 0.05).

A similar trend was found for leaf fresh weight and leaf area, with regressive *P. acutifolius* var. acutifolius being the type with the highest values compared to the others (*p* < 0.001). However, for cultivated *P. acutifolius* var. acutifolius, the values were higher for SLA and stomatal density compared to the other three types (*p* < 0.001). Regarding phenological behavior, the wild types of *P. acutifolius* var. acutifolius and *P. acutifolius* var. tenuifolius took 30 and 37 days after sowing, respectively, to reach flowering, contrary to what was presented for *P. acutifolius* var. acutifolius, both for cultivated and regressive types, which took between 35 and 42 days after sowing to reach flowering, respectively. This situation was contrary to that presented for physiological maturity, where all three types reached this phenological stage around 79 days after sowing. Pollen viability (PV) values ranged between 77 and 88%, where the *P. acutifolius* var. acutifolius regressive type had the lowest values (Figure 3). When we analyzed the differential of ambient temperature in relation to leaf temperature, we found that all the three types were below the ambient level, being more efficient in the dissipation mechanism by cultivated type of *P. acutifolius* var. acutifolius.

### 2.3. Fluorescence and Images of Chlorophyll a (Chl_a_) Fluorescence Parameters

**Figure 4:**
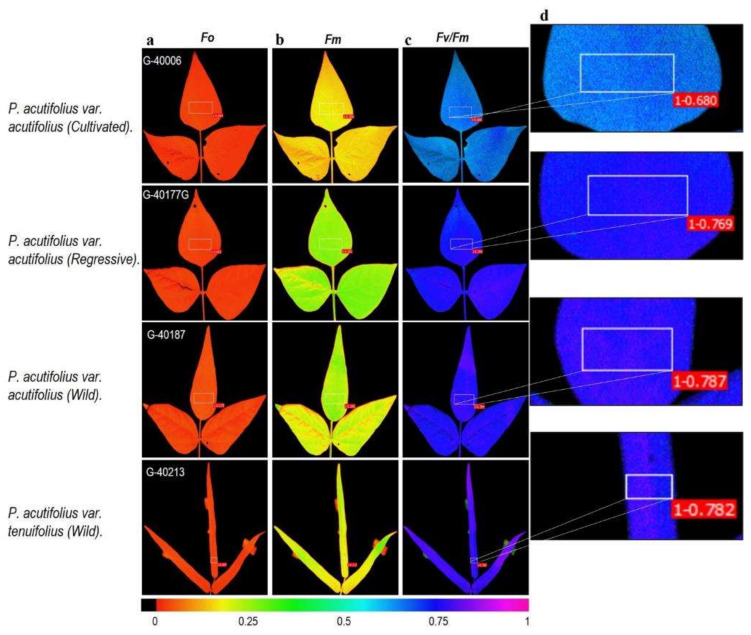
Images of chlorophyll a fluorescence parameters taken in fully developed leaves at the onset of physiological maturity the tepary bean genetic resource: (**a**) Initial fluorescence (F_0_), (**b**) maximum fluorescence (F_m_), (**c**,**d**) maximum quantum efficiency of PSII (F_v_/F_m_). (**d**) An example of the area of interest (AOI) tested on each leaf to obtain the fluorescence variables is shown. Gradient in color change from violet to red means higher to lower value for each variable.

Results on the quantitative analysis and images of Chl_a_ fluorescence parameters showed that the maximum quantum yield of PSII (F_v_/F_m_) was between 0.68 and 0.81 in the different accessions of tepary bean, the values being lower in the accessions of the cultivated type of *P. acutifolius* var. acutifolius (Figure 4). The regressive tepary accessions presented a higher magnitude of F_m_ values; however, the magnitude in F_0_ values were similar in the cultivated and regressive types of accessions, the lower values being more evident in the wild type of tepary tenuifolius beans (Figure 4).

**Figure 5:**
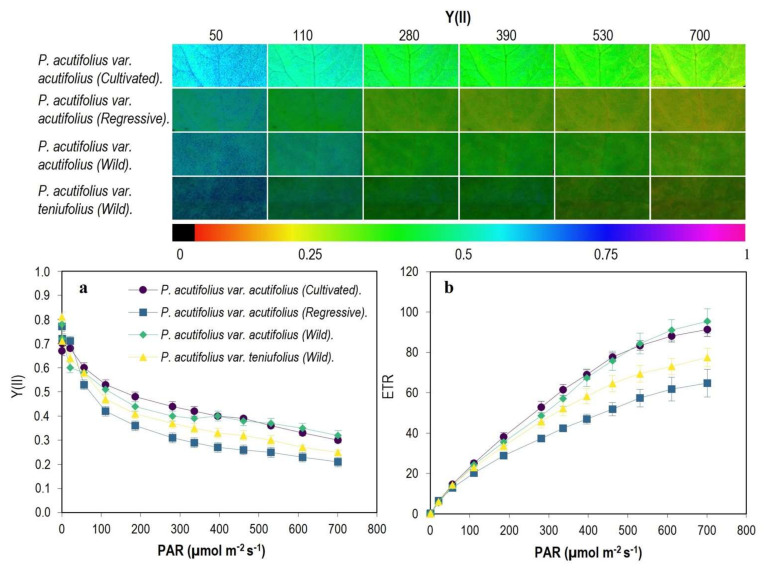
Behavior of the photosynthetic machinery as a function of PAR among the tepary bean genetic resource. (**a**) Photochemical yield (Y(II)), (**b**) electron transport rate (ETR). In the upper panel, gradient in color change from violet to red means higher to lower value for each variable. Means and error bars correspond to 20 AOI (area of interest) from four leaves of each accession, i.e., 5 AOI were tested on each leaf.

**Figure 6:**
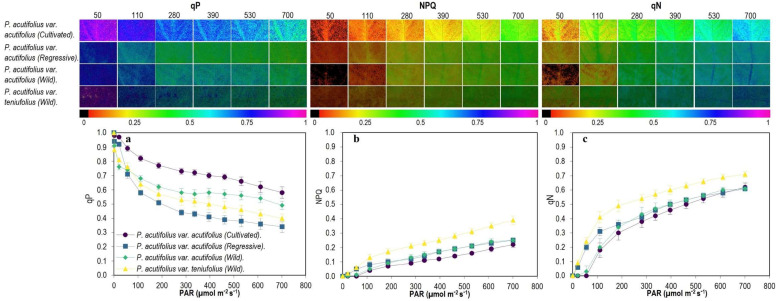
Different energy pathways as a function of PAR among the tepary bean genetic resource. (**a**) Photochemical quenching (qP), (**b**) non-photochemical quenching (NPQ), (**c**) coefficient of non-photochemical dissipation (qN). In the upper panel, gradient in color change from violet to red means higher to lower value for each variable. Means and error bars correspond to 20 AOI (area of interest) from four leaves of each accession, i.e., 5 AOI were tested on each leaf.

In general, we found that the photochemical yield (Y(II)), decreased with increasing PAR (Figure 5), the slope being steeper in the regressive type of *P. acutifolius* var. acutifolius accessions than the cultivated and wild types of *P. acutifolius* var. acutifolius, a situation that was also reflected in the electron transport rate (ETR; Figure 5). However, when we analyzed the energy use that is distributed specifically to the biochemical pathway (qP; Figure 6), we found that cultivated *P. acutifolius* var. acutifolius had much higher values compared to the other two types due to a higher fraction of open PSII centers (qL). Energy dissipation by non-photochemical quenching (NPQ) pathways, specifically in the form of heat increased proportionally with PAR starting at 300 μmol m^−2^ s^−1^, with the regressive *P. acutifolius* var. acutifolius type and wild type of *P. acutifolius* var. tenuifolius presenting the highest proportion in this pathway, and a similar behavior was also observed with the coefficient of non-photochemical dissipation (qN; Figure 6).

### 2.4. Correlations between Grain Yield and Agronomic and Physiological Variables under Temperature and Acid Soil Conditions

Traits, both agronomic and physiological, that are specific to each type of accessions were found in response to high temperature and acid soil stress conditions that influenced grain yield (GY; Table 1). We found variables that were expressed in only one type of accessions, such as height (r = 0.20) and stem biomass (r = 0.31), as well as pods (r = 0.25) and non-viable seeds (r = 0.18) per plant, specifically in cultivated type of *P. acutifolius* var. acutifolius, which showed a positive effect on GY. Likewise, the number of branches (r = 0.45) positively affected GY in the wild type *P. acutifolius* var. tenuifolius, but for this same wild type, pollen viability (r = −0.33) was negatively correlated with GY, probably due to soil acidity stress. There were other agronomic characteristics at the pod level (quantity and biomass as the number of viable pods) and at the seed level (length and width as seed biomass) that had a positive effect on GY in most of the three types. At the physiological level, we found that increasing the energy pathway in the form of heat (NPQ) decreased GY in all three types, being more evident in wild type of *P. acutifolius* var. acutifolius (r = −0.32). However, some types were more efficient in electron transport rate (ETR), which positively influenced the GY trait.

**Figure 7:**
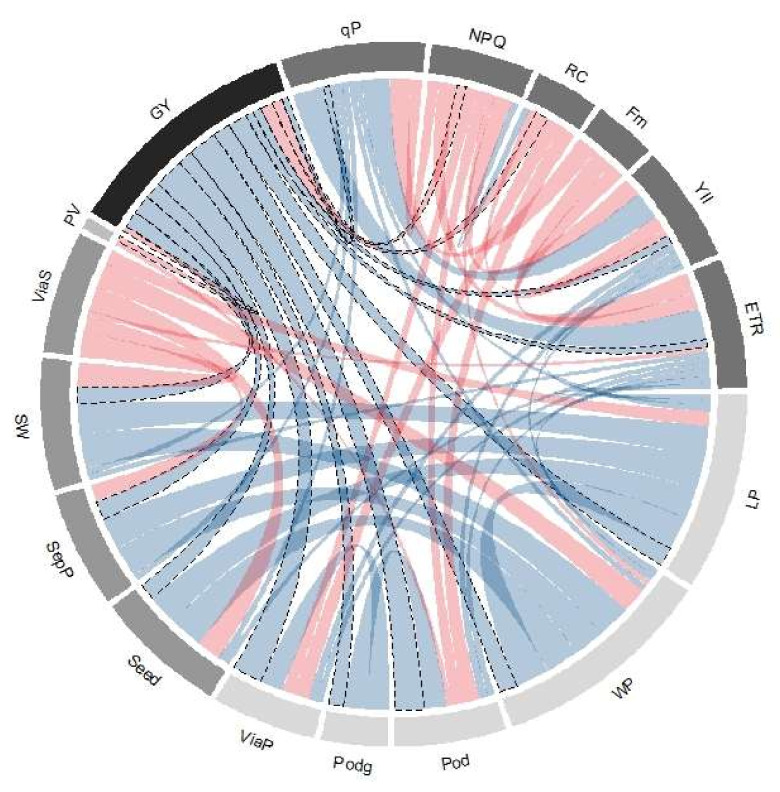
Chord diagram of correlation coefficients between agronomic, phenological and physiological variables the tepary bean genetic resource. Ribbons inside the circle correspond to significant correlations with a *p*-value < 0.05; blue ribbons indicate positive coefficients and red ribbons indicate negative coefficients. Width of pods (WP), length of pods (LP), pods mass (Podg), viability of pods (ViaP), seeds per pod (SepP), weight (g) of 100 seeds (SW), viability of seeds in the pod (ViaP), pollen viability (PV), grain yield (GY), photochemical pathway (qP), fraction dissipated as heat (NPQ), relative chlorophyll (RC), maximum fluorescence level (F_m_), photochemical yield (Y(II)), and electron transport rate (ETR).

Figure 7 shows the correlations between GY and the other agronomic, phenological, and physiological variables of *Phaseolus acutifolius* accessions. We found that GY had a high correlation with both number (Pod, r = 0.73) and viability of pods (ViaP, r = 0.72), as well as number of seeds per pod (SepP, r = 0.47). At the phenological level, a negative correlation was found with pollen viability (PV, r = −0.14), as well as with different physiological variables, such as heat energy dissipation (NPQ, r = −0.14). However, we found that GY in tepary accessions is correlated with energy directed to the photosynthetic machinery (qP, r = 0.15), as well as with PSII quantum efficiency (Y(II), r = 0.21, ETR, r = 0.19).

## 3. Discussion

### 3.1. Contribution of Morpho-Agronomic Traits of Tepary Bean for Adaptation to Combined Stress of High Temperature and Acid Soil

Differences among different phenotypic responses of a species reflect adaptations to the changing environment; thus, variation in plant traits and the degree of phenotypic plasticity is expected [35,36]. In this regard, tepary beans have been characterized as more tolerant to various abiotic and biotic stresses (high temperature, drought, diseases) than common beans [20,37], making them valuable genetic resource for improving stress tolerance through conventional breeding programs [38,39]. However, research on their adaptation has been focused on the areas where their domestication has been developed [21], and this has had an impact on the lack of information on their adaptation and response to other types of stresses, such as acid soils.

The results from this study showed significant variation in traits among the four evaluated types of tepary bean resulting from domestication of the germplasm [40,41]. For example, in terms of biomass, two wild types, *P. acutifolius* var. (wild) and *P. acutifolius* var. tenuifolius (wild), had lower main stem weight, with indeterminate growth habit with short guide compared to the other two types, *P. acutifolius* (cultivated and regressive), which showed numerous secondary and tertiary stems with a more prostrate indeterminate growth habit with climbing ability [42,43].

Likewise, root weight values showed marked difference between *P. acutifolius* (wild) and *P. acutifolius* tenuifolius (wild) in comparison with *P. acutifolius* (cultivated and regressive). A possible explanation for the difference in root biomass is due to the effect of soil acidity on root development specifically in wild accessions compared to cultivated ones [33,42,44]. On the other hand, other agronomic traits, such as pod size and seed weight, were highlighted in tepary accessions *P. acutifolius* var. (cultivated) and *P. acutifolius* var. (regressive), as they were seeds of larger size and with diverse colors (white, yellow, mottled) and with longer pods [5], which have been the product of strong selection pressure in the domestication process [45], and these characteristics distinguish cultivated from wild forms [46]. However, the viability of pods and seeds was found to be higher in wild *P. acutifolius* var. and *P. acutifolius* tenuifolius accessions with respect to *P. acutifolius* var. (cultivated) and *P. acutifolius* var. (regressive) types.

This behavior of *P. acutifolius* accessions could be interpreted in two ways; on the one hand, (i) the viability of the pod and seed in *P. acutifolius* accessions could indicate an expression of agronomic traits as a function of genotypic variability and heritability estimates, which may vary due to the genetic structure of each accession (wild versus cultivated) [47]. On the other hand, (ii) the degree of adaptation or susceptibility to environmental conditions, such as increased temperature, relative humidity, nutrient availability [40,48], rainfall, and low soil fertility which, as previously observed with common bean, could be a factor that influence both pod and seed viability [10,21,49,50]. Because of the above, most of the wild accessions of *P. acutifolius* and *P. tenuifolius* have the characteristics of producing small seed, which allows giving them an added advantage in grain filling and also with higher seed number, allowing better responses and less penalty in pod and grain formation under a stress condition [50].

In producing grain yield, the low variability presented between cultivated and wild accessions was marked more by phenotypic variation (response to the environment) than by genotypic variation (genetic constitution), suggesting that the accessions evaluated may share similar genetic backgrounds, which could be probably due to the localization center of their domestication, which may be considered as a genetic bottleneck [26]. This may have partly influenced the low genetic variation observed in this study and may have limited the genetic background shown by tepary bean confirming results from previous studies [26,41,51,52,53]. In this regard, our results suggest that possibly within the wild accessions are the parents of the cultivated accessions, due to similarities in the traits evaluated. We highlight the response of 25 accessions of *P. acutifolius* var. acutifolius: 6 cultivated *P. acutifolius* accessions (G 40271, G 40019, G 40006, G 40230, G 40012, G 40129) and 19 accessions of *P. acutifolius* tenuifolius (G 40222, G 40226, G 40253A, G 40221, G 40258A, G 40193, G 40224, G 40197, G 40220, G 40253, G 40261, G 40254, G 40256, G 40248, G 40210, G 40195A, G 40181, G 40289, G 40194) that showed higher values of both morphological and agronomic traits (higher than wild accessions), such as biomass of their vegetative organs, 100-seed weight, number of pods per plant, number of seeds per pod, and grain yield values of above 1800 kg ha^−1^. These attributes are important for the selection of adaptive traits to high temperature and acid soil conditions, and these are complementary for breeding, population development, and effective improvement of tepary bean [41,54]. Likewise, these traits can influence the response to selection of these accessions and can also serve as selection criteria for the improvement of common bean [55].

### 3.2. Leaf Characteristics and Phenological Development Contribute to Better Adaptation of Tepary Beans to Combined Stress

Morphologically, cultivated, and regressive accessions are different from wild accessions [26,56]. Cultivated *P. acutifolius* var. acutifolius have sub-ovate to oval-shaped leaflets, while wild *P. acutifolius* var. acutifolius have sub-ovate triangular shaped leaflets, and *P. acutifolius* var. tenuifolius are characterized by narrow and linear leaflets [57]. This difference in leaflet shape, size, and leaf arrangement impacted the traits assessed at the leaf level in our study (leaf weight, leaf area and specific leaf area, and stomatal density). For example, cultivated *P. acutifolius* var. acutifolius developed leaflets with greater leaf area than the other accessions and exhibited higher stomatal density, suggesting greater CO_2_ absorption and water loss, traits that favor them with better photosynthetic capacity and greater leaf development [58,59,60]. The low stomatal density (SD) presented by wild accessions, specifically in *P. acutifolius* tenuifolius accessions, is a characteristic of species adapted to drought conditions and sites with water deficits (more conservative use of water, with limitations to some extent in stomatal conductance and transpiration) that results in a lower proportion of dry matter but with a high amount of seeds per plant [61,62,63,64]. However, under opposite conditions, such as those presented in the Amazon (high humidity and acidic soils), the decrease in SD in most wild accessions may be attributed to soil acidity accompanied by high rainfall and high temperature [60,65,66], without compromising plant development and growth [64,67]. Therefore, based on our results that relate to differences in leaf shape, such as SD, between cultivated and wild accessions could be a product of adaptation to local environmental conditions. This underscores the gene pool differences observed in tepary bean, and, specifically in the degree of phenotypic plasticity, they exhibit to adapt to or tolerate change in environmental conditions [52].

When analyzing phenology in tepary accessions, we found that they tend to present earliness interms of days to flowering (DF) and days to physiological maturity (DMP). We found wild *P. acutifolius* and wild *P. tenuifolius* were early at both growth stages, and this is a characteristic trait of their adaptation to high temperature conditions [68]. However, from the data obtained in this study, the wild accessions presented early flowering that resulted in early maturity, which resulted in reduced dry matter production, as well as number of pods per plant [54]. Thus, we speculate that phenological response difference among the evaluated tepary bean accessions could be due to genetic rather than environmental factors [69], with wild accessions being more affected than cultivated ones.

Studies on pollen viability under humid tropical climatic conditions resulted in identification of common bean genotypes that are adapted to stress induced by high temperatures [10,70]. Naturally, in our evaluations, the high ability of tepary beans to adapt to high temperature conditions allowed normal growth and development without heat stress-induced effects [17,41], demonstrating the development of avoidance mechanisms resulting from selection and domestication [45,71].

Our results indicate that the combination of phenological attributes, such as flowering and early maturity, with agronomic attributes, such as higher number of pods per plant and grain yield, shown by the accessions of *P. acutifolius* var. acutifolius cultivated type (25 accessions), makes these tepary bean accessions as valuable genetic resources with potential to boost production in the Amazon region. These accessions can contribute to develop new tepary bean populations [41] and also could serve as a source of valuable genes to improve abiotic stress tolerance of common bean [54,72].

### 3.3. Energy Dissipation Mechanisms in Response to High Temperature and Acid Soil Stress

Generally speaking, *P. acutifolius* accessions responded to environmental conditions by activating regulatory mechanisms to balance or adjust PSI and PSII yield to protect the photosynthetic apparatus [73,74], and such adjustments are necessary mainly because photosystem II (PSII) is very sensitive to environmental stress and it plays a key role in the response of photosynthesis to environmental perturbations, including aluminum (Al) toxicity [75,76]. For example, we observed that F_0_ and F_m_ values varied slightly over time; however, these variations, at the accession level, only presented effect on PSII yield in some cultivated *P. acutifolius* accessions, causing decreases in F_v_/F_m_ (0. 68). Possibly, this decrease is related to reversible changes in electron flow and heat dissipation used to adjust PSII quantum efficiency, avoiding photoinhibitory damage [77,78]. This would indicate that at the level of PSII photochemistry the cultivated *P. acutifolius* var. acutifolius accessions have a relatively adequate capacity to cope with soil acidity as stress progresses and, therefore, are able to show a better physiological state of their photosynthetic apparatus [75,79].

We observed that a higher proportion of incident radiation was allocated to PSII photochemistry (ΦPSII), in wild-type *P. acutifolius* var. acutifolius and cultivated *P. acutifolius* accessions, indicating a higher proportion of open reaction centers [80] and higher flux in electron transport (ETR) [81]. However, the strong decreases of qL and qP in regressive *P. acutifolius* var acutifolius and wild-type *P. acutifolius* var. tenuifolius accessions could be an indication that PSII activity was limited by the accumulation of quinone A (Q_A_) electrons, implying that electron transfer was blocked at a step after plastoquinone reduction, being likely on the acceptor side of PSI [82]. Likewise, the steady decrease in qP and qL as PAR increased suggests that it possibly became progressively more restrictive with fluctuating light [83]. This inhibition of light-dependent reactions was accompanied by an increase in NPQ and qN, as possible mechanisms of energy dissipation and/or adaptation [74]. We found that NPQ and qN increased under acidic soil, indicating a significant increase in thermal dissipation to compensate for reduced photochemical dissipation. We demonstrated a significant increase of both NPQ and qN that could have greater protection function against photoinhibition as photoprotective capacity against oxidative damage in regressive *P. acutifolius* var. acutifolius and wild *P. acutifolius* tenuifolius accessions [84,85,86].

### 3.4. Adaptive Mechanisms for Increasing Grain Yield under Combined Stress of High Temperature and Acid Soil

A high correlation between two tested traits suggests that selection for one trait will cause a change through additive genetic effects of selected accessions and simultaneously a direct change in the other trait [54]. Looking further into the correlations between responses across the four *P. acutifolius* types evaluated, we found that the traits most associated for increased adaptation under acid soil conditions are related to agronomic traits, such as pod and seed weight, number, and viability. In addition, the characteristic of producing small seeds could be an advantage over common bean, which can be interpreted as a compensatory trait under a stress condition and which indicates that selection for these traits can advance genetic gain in tepary bean breeding [41,50,54,69]. At the physiological level, we observed that part of the adaptive traits shown by the types of *P. acutifolius* are toward a greater allocation of energy in non-photochemical pathways (NPQ and qN), a feature that allows them to have a more negative LTD (i.e., the leaf is cooler than the air temperature). This suggests that it is related to greater heat dissipation to compensate for reduced photochemical dissipation, reducing water use, keeping stomata open for CO_2_ diffusion, thus enhancing transpiratory cooling, which allows the leaves of these accessions to reach temperatures below −6 °C relative to ambient temperature without compromising photosynthetic machinery and GY [5,21,39,84,87]. Results from the present study indicate that selection based on morpho-agronomic and physiological attributes could contribute toward improving yield gains in *P. acutifolius* breeding programs, and the identified, stress adapted accessions could also serve as a valuable genetic resource for improving common bean yield under the combined stress conditions of high temperature and acid soil.

## 4. Materials and Methods

### 4.1. Experimental Site and Meteorological Conditions

**Figure 8:**
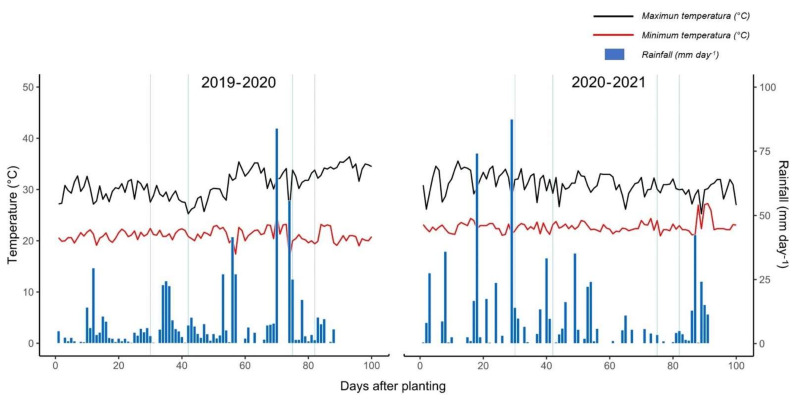
Distribution of rainfall and maximum/minimum temperatures during the crop growing period at the Macagual Research Center in Colombia in two seasons: October 2019–January 2020 (**left**); and October–January 2021 (**right**).

A total of 302 tepary bean accessions (162 *P. acutifolius* var. acutifolius cultivated; 6 *P. acutifolius* var. acutifolius regressive; 61 *P. acutifolius* var. acutifolius wild; 73 *P. acutifolius* var. tenuifolius wild) were evaluated at the Macagual Research Center of the Universidad de la Amazonia, Colombia (1°37′ N and 75°36′ W). The Center is located in the municipality of Florencia, Caquetá (Colombia), in a tropical rainforest ecosystem. The average annual rainfall is 3800 mm, with an average temperature of 25.5 °C and a relative humidity of 84% and a sunshine of 1700 h of sunshine per year. The evaluations were conducted in two seasons ((i). October 2019 to January 2020; (ii). October 2020 to January 2021) during the minimum precipitation period of the year, period that corresponds to the driest months in which air temperature is higher compared to the other months of the year, in which average minimum and maximum temperature ranged between 20.24 to 28.10 °C and 22.7 to 31.01 °C; and total precipitation values during the first and second season were 471.3 and 684.2 mm, respectively (Figure 8).

### 4.2. Plant Material and Experimental Design

A total of 302 *Phaseolus acutifolius* accessions from two different varieties (acutifolius and tenuifolius) and four different types of tepary bean genetic resource (*P. acutifolius* var. acutifolius cultivated, *P. acutifolius* var. acutifolius regressive, *P. acutifolius* var. acutifolius wild, *P. acutifolius* var. tenuifolius wild), that were collected from different countries, were used and seeds were supplied by the genetic resource bank of the International Center for Tropical Agriculture (The Alliance Biodiversity International and CIAT), and these genetic resources were evaluated in this study (Supplementary material Table S1 [27]). A completely randomized block design with three replications was used for the evaluation, and the randomized plots within the block were each accession of *Phaseolus acutifolius*. Ten plants were planted in each plot for a total of 9060 plants monitored. Each seed was sown in a 3 L bag whose soil used for the evaluation was characterized by pH values ranging between 4.1 and 5.2, with high aluminum saturation (73.4%) and exchangeable aluminum content of 6.3 cmol kg^−1^, and exchangeable soil acidity up to 1.98 cmol kg^−1^. The organic matter content was very low (1.35%) as was the available phosphorus (2.58 mg kg^−1^, Bray-II) with a total base saturation of 7.1% (cmol kg^−1^: Ca: 0.38, Mg: 0.1, K: 0.14, Na: 0.1, total bases: 0.8) and a cation exchange capacity of 11.3 cmol kg^−1^.

### 4.3. Development of Different Vegetative Organs under High Temperature and Acid Soil Stress Conditions

At the time of harvest, different plant organs were counted, measured, and weighed. The number of developed branches was counted and the pods, stem, and root system parts were weighed for each plant. The length and width of the pod and of the harvested seeds of each replicate (plant) per each tepary accession were measured. The number of viable and non-viable pods and seeds per plant was quantified, as well as the number of viable and non-viable seeds per pod. For each accession, 100 seeds (SW) were randomly selected to determine seed weight (g), and grain yield (kg ha^−1^) was determined by threshing and cleaning the pods of harvested plants.

### 4.4. Morphological and Phenological Characteristics

At mid-pod filling growth stage, leaves were collected per plant in each tepary accession (*n* = 100), fresh weight was determined, and leaf area and specific leaf area (SLA) were measured. Each leaf was weighed independently using an Ohaus Scout electronic balance (100 ± 0.001 g), after which they were scanned using the HP ScanJet Pro 2500 scanner (Palo Alto, CA, United States) to determine leaf area using ImageJ software (Annapolis, MD, United States). Leaf discs of known area (3.14 cm^2^) were oven dried and weighed to determine SLA. Stomatal density (number of stomata per unit area) (SD) was determined from the impression obtained on the adaxial surface of the leaf using transparent enamel, which was taped to a slide. Four leaves on four plants per Tepary accession were used for a total of 4832 units monitored. For each slide reading, two random counts were made to determine the density of stomata (number mm^2^). Likewise, the number of days to flowering (DF) and days to physiological maturity (DPM), as well as pollen viability (PV), as described by Suárez et al. [10,19,34], were measured in order to determine the effects on phenological traits of different tepary accessions. The leaf temperature differential (LTD) corresponding to the difference between leaf and ambient temperature was measured using the MultispeQ handheld device [88] (Lansing, MI, USA).

### 4.5. Fluorescence and Imaging for Chlorophyll (Chla) Parameters

From the color gradients and trends obtained with increasing photosynthetically active radiation (PAR) obtained from the Imaging-PAM M-Series and the software version 2.32 Imaging WIN (Heinz Walz GmbH, Effeltrich, Germany), different physiological parameters related to chlorophyll fluorescence (Chl_a_) were determined on fully developed leaves (*n* = 16) at the onset of physiological maturity. In the dark, after initial adaptation to darkness for 60 min, the initial fluorescence level (F_o_) was determined, and, after applying a pulse of actinic light, the maximum fluorescence level (F_m_) was determined. After the application of continuous actinic light saturating pulses, the fluorescence level increases to the Fm level up to the Fm′ level, thereby determining the amount of energy taken by the photochemical pathway (qP = (F_m_′ − F)/(F_m_′ − F_o_′)) [89]. Likewise, based on the equations proposed by Kramer et al. [90], the other routes that energy can take were determined, and these include the fraction of photoprotection induced by light through thermal dissipation of energy ((NPQ) = (F_s_/F_m_′) − (F_s_/F_m_)) and the fraction not regulated by other non-photochemical losses ((qN) = F_s_/F_m_). The maximum PSII quantum yield (Fv/Fm) and the apparent electron transport rate (ETR) [91,92], as well as the photochemical yield ((YII = (F_m_′ − F_o_)/F_m_′)), were calculated as proposed by Kramer et al. [90]. Images were taken on fully developed leaves at the vegetative developmental stage of flowering and pod filling, following the methodology described by Ríos et al. [93], to capture Chl_a_ fluorescence emission transients.

### 4.6. Data Analysis

An analysis of variance was carried out to evaluate the significant differences in each of the morphological, phenological, agronomical and physiological variables among four different types of tepary bean genetic resource (cultivated, regressive, wild, and wild) of *Phaseolus acutifolius* accessions. For this purpose, a Linear Mixed Model (LMM) was fitted to analyze the effect of the fixed factor (tepary bean genetic resource). The plots associated with the accessions within the block in the monitoring period were included as random effects (repeated measures). The assumptions of normality and homogeneity of variance were evaluated by means of an exploratory analysis of residuals. By using a Fisher’s LSD post-hoc test (α = 0.05), differences among tepary bean genetic resources were analyzed. Using the results from the analysis of variance, box plots were prepared to show the differences for each of the variables tested. Pearson correlation analysis was used to determine the relationship between grain yield and the other morpho-agronomic and physiological variables for each type of tepary bean genetic resource. Correlations were shown in a general way by means of using a chord diagram including the packages of corrplot [94] and circlize [95]. The LMMs were performed in the R language software, version 3.4.4 [96], using the interface in InfoStat, with the lme function of the nlme package, and the graphical outputs were performed in the packages of “ade4”, “ggplot2”, “factoextra”, and “corrplot”. [97]

## 5. Conclusions

In this study, we evaluated the adaptation of 302 accessions of tepary bean and its wild relatives under the combined stress conditions of high temperature and acid soil in the Amazon region of Colombia. We found significant differences among four types of tepary bean genetic resource in terms of their phenological, morpho-agronomic, and physiological responses to combined stress conditions. We have identified six cultivated type of accessions of *P. acutifolius* var. acutifolius and 19 wild type of accessions of *P. acutifolius* tenuifolius that exhibited grain yields above 1800 kg ha^−1^. Several of these accessions could be suitable to use as parents to improve dry seed production of tepary bean under combined stress conditions of high temperature and acid soil.

## Figures and Tables

**Table 1:** Correlation coefficients between grain yield (GY) and different agronomic and physiological traits of the tepary bean genetic resource grown under acid soil and high temperature conditions.

Trait	*P. Acutifolius var. Acutifolius (Cultivated)*	*P. Acutifolius var. Acutifolius (Regressive)*	*P. Acutifolius var. Acutifolius (Wild)*	*P. Acutifolius var. tenuifolius (Wild)*
Plant height at 10 days	0.20 *	0.17	0.12	0.12
Number of branches/plant	−0.15	−0.31	0.10	0.45 ***
Stem weight/plant (g)	0.31 ***	0.06	−0.03	0.07
Fresh Weight (g)	−0.04	−0.01 **	0.32	−0.04
Total number of pods/plant	0.84 ***	0.63	0.65 ***	0.62 ***
Pod weight/plant (g)	0.41 ***	0.73 **	0.47 ***	0.34 **
Number of viable Pods/plant	0.84 ***	0.61	0.68 ***	0.63 ***
Number of non-Viable pods per plant	0.25 **	0.28	0.18	0.03
Width of pods (mm)	0.36 ***	0.49	0.62 ***	0.67 ***
Length of pods (mm)	0.41 ***	0.53	0.59 ***	0.62 ***
Seed weight/plant (g)	0.35 ***	0.88 ***	0.67 ***	0.73 ***
Number of viable seeds (No)	−0.04	−0.27	−0.26 *	−0.37 **
Number of seeds per pod (g)	0.42 ***	0.78 **	0.70 ***	0.76 ***
Number of viable seeds per plant	0.77 ***	0.37	0.59 ***	0.47 ***
Number of non-viable seeds per plant	0.18 *	0.10	0.11	0.06
100 seed weight (SW, g)	0.35 ***	0.88 ***	0.67 ***	0.73 ***
Relative chlorophyll (RC)	−0.24 **	0.03	−0.03	−0.27 *
Pollen Viability (PV, %)	0.14	−0.24	−0.14	−0.33 **
F_m_′	−0.11	0.07	0.30 **	0.14
Y(II)	0.11	0.22	0.24	0.28 **
NPQ	−0.18 *	−0.09	−0.32 **	−0.30 **
qN	−0.10	0.03	−0.35 **	−0.24 **
ETR	0.12 ***	0.50	0.15	0.26 **

Maximum fluorescence level (F_m_′), photochemical yield (Y(II)), fraction dissipated as heat (NPQ), fraction not regulated by other non-photochemical losses (qN), and electron transport rate (ETR). Mean values were used in the correlation analysis, and *, **, and *** represent probability levels of significance of 0.05, 0.01, and 0.001, respectively.

## Data Availability

Data are available from the authors upon request.
